# Trends in Informal and Formal Care Use and Unmet Needs among Older Population, 2014-2023

**DOI:** 10.1093/geroni/igaf122.2663

**Published:** 2025-12-31

**Authors:** Eun Ha Namkung, Gyeonghee Lee

**Affiliations:** Ewha Womans University, Seoul, Republic of Korea; Ewha Womans University, Seoul, Republic of Korea

## Abstract

The increasing number of older adults with limitations in daily activities has significant implications for policy and social systems, particularly in rapidly aging societies. Recognizing that how care needs are met depends on both individual characteristics and societal changes, this study examines trends in informal and formal care utilization and their influencing factors among older Koreans over the past decade. Using data from four waves of the National Survey of Older Koreans (2014, 2017, 2020, and 2023), we analyze changes in care received by community-residing adults aged 65 and older with care needs (n = 1,153 to 2,902 per wave). Survey-weighted multinomial regression models show a rising proportion of older Koreans with unmet care needs, increasing from 20.8% in 2014 to 52.6% in 2023. Informal care from family and friends has declined sharply, while the modest rise in formal care use has not offset the growing care gap. Lower economic status is consistently linked to higher unmet care needs, while older age, living alone, and greater activity limitations are associated with formal care use. These findings highlight the urgent need for expanding formal care services and supporting informal caregivers. Further research is needed to understand the societal and policy shifts contributing to rising unmet care needs and to inform interventions that improve access to care for vulnerable older adults.

